# Thyroid gland cutaneous fistula secondary to a migratory fish bone: a case report

**DOI:** 10.1186/1752-1947-6-140

**Published:** 2012-06-01

**Authors:** Toyoaki Ohbuchi, Takahisa Tabata, Khac-Hung Nguyen, Jun-ichi Ohkubo, Akiko Katoh, Hideaki Suzuki

**Affiliations:** 1 Department of Otorhinolaryngology, School of Medicine, University of Occupational and Environmental Health, Kitakyushu, Japan

## Abstract

**Introduction:**

We report an extremely rare case of a migratory fish bone penetrating through the thyroid gland.

**Case presentation:**

A 56-year-old Japanese woman presented with a two-month history of a painless cutaneous fistula in her anterior neck with pus discharge. Endoscopic examinations showed no abnormality, but computed tomography revealed a bone-density needle-shaped foreign body sticking out anteroinferior from the esophagus wall, penetrating through her left thyroid lobe and extending nearly to the anterior cervical skin. A migratory fish bone was suspected, and the foreign body was removed under general anesthetic, combined with a hemithyroidectomy. The injured esophageal mucosa was sutured and closed. Our patient’s postoperative course was uneventful, and she was allowed oral food intake seven days after the surgery. No evidence of recurrence was seen over the postoperative follow-up period of 42 weeks.

**Conclusion:**

We should be aware that fish bone foreign bodies may migrate out of the upper digestive tract and lodge in the thyroid gland.

## Introduction

Fish bones are one of the commonest foreign bodies occurring in the pharynx or cervical esophagus. Fish bone foreign bodies located outside the aerodigestive tract are relatively rare. Here, we present an extremely rare case of a migratory fish bone penetrating through the thyroid gland with a cutaneous fistula in the anterior neck one year after swallowing the foreign body.

## Case presentation

A 56-year-old Japanese woman had visited a local clinic, complaining of a two-month history of a painless cutaneous fistula in her anterior neck with pus discharge. She was diagnosed with a pyogenic granuloma [1] by her previous doctor. The pus discharge temporarily subsided after antibiotic treatment, but the cutaneous fistula persisted. She was then referred to our department for further advice. On physical examination, a non-tender reddish lesion with a fistula and granulation was seen on her anterior cervical skin. Our patient reported that she had suffered from the sensation of a foreign body in her throat after eating fish approximately one year prior to the present onset, but the symptom had spontaneously improved after a few days. Taking her anamnesis into account, a fish bone foreign body was suspected.

**Figure 1:**
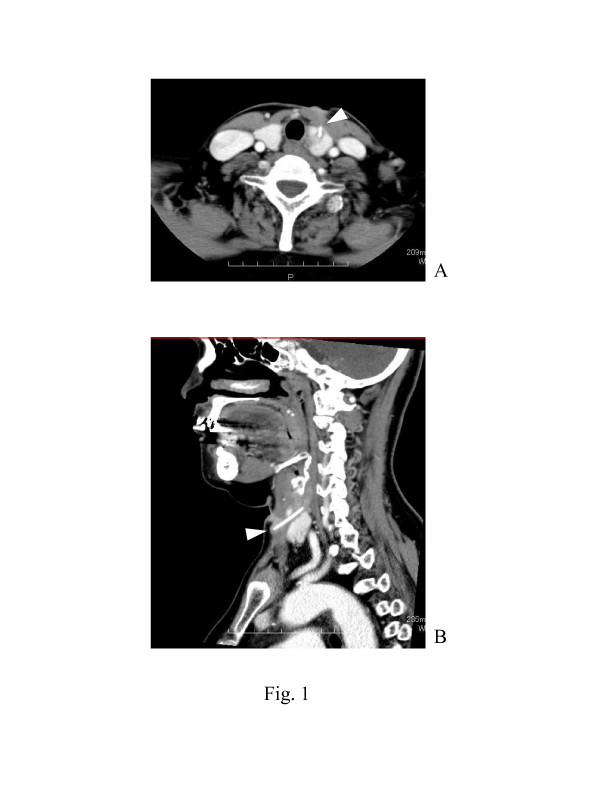
** Preoperative computed tomography findings.** Contrast-enhanced **(A)** axial and **(B)** sagittal computed tomography reveal a bone-density needle-shaped foreign body sticking out anteroinferior from the esophageal wall, penetrating through the superior pole of the left thyroid lobe, and extending nearly to the anterior cervical skin. The foreign body is surrounded by a low density area, suggesting abscess formation.

Endoscopic examinations of her laryngopharynx and esophagus showed no abnormality. However, computed tomography (CT) revealed a bone-density needle-shaped foreign body sticking out anteroinferior from the esophageal wall, penetrating through the superior pole of her left thyroid lobe, and extending nearly to the anterior cervical skin (Figure 1). The foreign body was surrounded by a low density area, suggesting abscess formation (Figure 1). The results of a blood examination were unremarkable, with normal thyroid and parathyroid functions.

**Figure 2:**
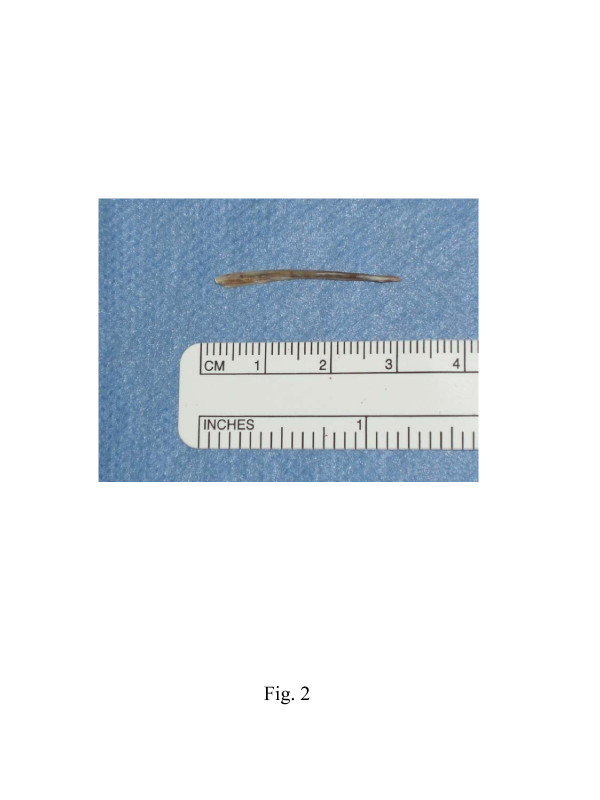
** Removed fish bone. **The removed fish bone is 28mm in length.

Our patient underwent the removal of the foreign body combined with left hemithyroidectomy under general anesthesia 20 days after her initial visit to our department. A horizontal cervical incision with the spindle-shaped excision of the orifice of the fistula was made, and the skin flap was elevated. Considerable cicatricial adhesion was seen around the fistula. The fistula passed through the infrahyoid muscles and was connected to her left thyroid gland. The tip of the fish bone was exposed by cutting her infrahyoid muscles open. As seen in the preoperative imaging, the fish bone penetrated through her thyroid gland, reaching the esophageal wall. The fish bone, fistula, left thyroid lobe and an abscess were totally removed with preservation of her recurrent laryngeal nerve. The injured esophageal mucosa was sutured and closed. Figure 2 shows the removed fish bone, measuring 28mm in length.

Our patient’s postoperative course was uneventful. No esophageal leakage was observed during an esophagography, and she was allowed oral food intake seven days after the surgery. She was discharged nine days after surgery, and no evidence of recurrence was seen over the postoperative follow-up period of 42 weeks.

## Discussion

Migratory fish bone foreign bodies in the thyroid gland are extremely rare, and only five other cases of this event have been reported in the English language literature since 1990 [2-6]. Moreover, to the best of our knowledge, no previous report has documented the formation of a cutaneous fistula in the anterior neck caused by a fish bone foreign body penetrating the thyroid gland. It is known that fish bones may pass through the pharyngeal or esophageal wall spontaneously or during endoscopic manipulation. Even if the fish bone does not migrate out of the pharyngeal or esophageal lumen, the puncture wound created by the foreign body may cause a thyroid abscess [7-9]. In the present case, it is most likely that the fish bone stuck in her cervical esophagus spontaneously migrated out of the lumen anteriorly, penetrated through her esophageal wall and thyroid gland, and reached her anterior cervical hypodermis in the course of one year after she swallowed the fish bone.

Considering that extrapharyngeal and extraesophageal foreign bodies often cause severe complications, such as deep neck infection or abscesses, mediastinitis and even sepsis, the early diagnosis and prompt removal of the foreign body are very important. The definitive diagnosis of a fish bone foreign body is successfully made by CT, which can locate and depict an extrapharyngeal or extraesophageal fish bone as a very high density needle-shaped material. Ultrasonography is also a useful diagnostic tool because most solid foreign bodies show acoustic shadows [4]. In contrast, cervical plain film radiography is less useful, because fish bones on this imaging usually exhibit only faint shadows, and often overlap with the thyroid and cricoid cartilages and vertebrae. We therefore cannot exclude the possibility that a fish bone foreign body is present, even if it is not visible on plain film radiography [2]. Endoscopic examination is often useless as well for the diagnosis or exclusion of migratory foreign bodies. In the present case, endoscopic examination of the laryngopharynx and esophagus did not reveal any mucosal damage, but CT of her neck performed at the first presentation clearly depicted an extrapharyngeal or extraesophageal fish bone.

The only radical treatment for an extrapharyngeal or extraesophageal fish bone is complete removal of the foreign body. If the fish bone is stuck in the thyroid gland, hemithyroidectomy may be included. Any abscess lesion formed in the surrounding tissue must be drained or resected simultaneously [3]. In the present case, hemithyroidectomy and the resection of an abscess were performed in addition to the removal of the foreign body to prevent the exacerbation of infection. Because a previous report documented that more than 10 % of patients who had undergone hemithyroidectomy manifested hypothyroidism [10], we should monitor the patient’s thyroid function.

## Conclusions

We experienced an extremely rare case of an extrapharyngeal and extraesophageal migratory fish bone foreign body with a cutaneous fistula, removed one year after swallowing the fish bone. We should be aware that fish bone foreign bodies may migrate out of the upper digestive tract, lodge in the thyroid gland and reach the cervical skin.

## Consent

Written informed consent was obtained from the patient for publication of this case report and any accompanying images. A copy of the written consent is available for review by the Editor-in-Chief of this journal.

## Competing interests

The authors declare that they have no competing interests.

## Authors’ contributions

TO, TT and HS analyzed and interpreted the patient data. TO and HS were major contributors in writing the manuscript. All authors read and approved the final manuscript.
